# Hypoxia boosts pluripotent-like muse cell ratio in mesenchymal stromal cells and upregulates the pluripotency gene expression

**DOI:** 10.1038/s41598-025-03806-x

**Published:** 2025-08-25

**Authors:** Gen Li, Masaaki Kitada, Mari Dezawa

**Affiliations:** 1 Department of Stem Cell Biology and Histology, Tohoku University Graduate School of Medicine, 2-1 Seiryo-machi, Aoba-ku, Sendai, 980-8575 Miyagi Japan; 2 Department of Anatomy, Kansai Medical University School of Medicine, Hirakata, Osaka Japan

**Keywords:** Hypoxia, Hypoxia-inducible factor, Muse cells, Mesenchymal stromal cells, Pluripotency genes, Stem cells, Biochemistry

## Abstract

Muse cells are SSEA-3-positive pluripotent-like endogenous stem cells found in various tissues, including peripheral blood and organ connective tissue. Their reserve is considered the hypoxic bone marrow. In mesenchymal stromal cell (MSC) cultures, Muse cells comprise several percent of the population. Clinical trials using intravenous administration of Muse cells without genetic modification or differentiation induction have shown significant therapeutic potential. Since Muse cells are a small fraction of MSCs, developing efficient culture methods to increase their proportion while maintaining their stemness is crucial for enhancing efficiency and reducing costs in clinical research. In this study, we investigated the effects of hypoxia on Muse cell proportions, pluripotency gene expression, and metabolism. Hypoxia increased the Muse cell proportion around twofold, driven by HIF2α rather than HIF1α, and enhanced pluripotency gene expression, potentially via microRNA let-7 upregulation. Hypoxia also shifted metabolism from oxidative phosphorylation to glycolysis, linked to maintaining stem cell properties. These findings suggest that hypoxia represents a cost-effective strategy for expanding Muse cells, offering promising potential for clinical applications.

**Supplementary Information:**

The online version contains supplementary material available at 10.1038/s41598-025-03806-x.

## Introduction

The main reserve of pluripotent-like multilineage-differentiating stress-enduring (Muse) cells, endogenous reparative stem cells found in the peripheral blood and connective tissues of various organs as cells positive for pluripotent surface marker stage-specific embryonic antigen (SSEA)−3, is considered the bone marrow (BM)^1–6^. Muse cells are also contained in cultured mesenchymal stromal cells (MSCs) and fibroblasts as several percent of the total population^2,6^. Muse cells express pluripotency markers such as POU class 5 homeobox 1 (*POU5 F1*), SRY-box transcription factor 2 (*SOX2*), and Nanog homeobox (*NANOG*) at moderate levels compared to embryonic stem cells (ESCs) and induced pluripotent stem cells (iPSCs), while higher than general somatic cells such as fibroblasts^7^. They also exhibit trilineage differentiation and self-renewal at a single-cell level^2,8^.

While Muse cells exhibit pluripotent-like characters, they are non-tumourigenic and express low-level telomerase, consistent with the fact that they are endogenous to the body. The pluripotency gene expression in Muse cells does not depend on the oncogene LIN28, a key factor for maintaining pluripotency in ESCs and iPSCs. Still, it is regulated by the tumour-suppressor microRNA let-7 that inhibits the PI3 K-AKT pathway, relevant to differentiation initiation and maintains the expression of Kruppel-like transcription factor 4 (*KLF4*) and its downstream *NANOG*, *POU5 F1*, and *SOX2*^2^^[,9^. This unique mechanism allows Muse cells to retain their pluripotent-like state and non-tumourigenicity, rendering them less concerned for therapeutic applications.

Muse cells express the sphingosine-1-phosphate receptor (S1PR), enabling them to sense sphingosine-1-phosphate (S1P) generated from apoptotic/damaged cells. Upon detecting these signals, Muse cells migrate to the injury site, engulf apoptotic/damaged cell fragments by phagocytosis, quickly recycle factors necessary for differentiation such as transcription factors that were active in apoptotic/damaged cells, and differentiation into the same cell type as the apoptotic/damaged cells, thereby replacing apoptotic/damaged cells with healthy functional cells and repair the tissue^10,11^. This process allows Muse cells to contribute to daily damage repair and maintenance in the body. Due to an immune privilege system, allogenic-Muse cells escape immune rejection and can survive for an extended period without immunosuppressant^12^.

For these characteristics, intravenously injected Muse cells have demonstrated non-tumourigenicity and therapeutic effects in various animal models, including those for stroke, liver disease, and kidney disease^13–15^. Clinical trials all conducted by intravenous drip of donor-derived Muse cells without immunosuppressant treatment demonstrated the safety and therapeutic effects of Muse cells in acute myocardial infarction, subacute ischemic stroke, epidermolysis bullosa, amyotrophic lateral sclerosis, cervical spinal cord injury, and neonatal hypoxic-ischemic encephalopathy^16–22^. A treatment that does not require donor selection, surgical treatment, gene introduction, or differentiation induction can be provided by Muse cells.

A substantial number of Muse cells are required to meet the demands of clinical therapy. Clinical-grade Muse cells were produced by selecting Muse cells from human MSCs^19,20^. While Muse cells can maintain self-renewal within MSCs, their proportion in MSCs is only several percent, and the production of clinical-grade Muse cells requires a large number of MSCs. If we can identify an efficient method to increase the proportion of Muse cells within MSCs without employing techniques that might alter cell properties, such as gene introduction, it would be highly beneficial for clinical applications.

Unlike the atmospheric oxygen (O_2_) concentration of 21%, known as normoxia, the O_2_ levels in vivo vary among the tissues from over 10% to below 1%^23^. In the BM, where Muse cells reside, the O_2_ concentration is as low as ~ 1.3%^24^. Hypoxia has gained significant attention in stem cell research due to its crucial role in promoting self-renewal and maintaining stemness. For instance, in mouse muscle satellite stem cell-derived primary myoblasts, 1% O_2_ hypoxia enhances their self-renewal and reduces differentiation^25^. Similarly, 0.3% O_2_ hypoxia accelerates the proliferation of neural stem cells isolated from the rat subventricular zone^26^, and a hypoxic environment helps maintain the quiescence and stemness of haematopoietic stem cells (HSCs)^27–29^. Culturing MSCs under hypoxia conditions reduces reactive oxygen stress (ROS), thereby alleviating oxygen stress and promoting genetic stability^30^. Hypoxia has also been shown to inhibit MSC senescence^31,32^. The mammalian reproductive tract, where the embryo develops, is also hypoxic, with O_2_ levels ranging from 1.5–5.3%^33–35^. Hypoxia has also been shown to maintain the pluripotency of human ESCs and promote the reprogramming of embryonic fibroblasts into iPSCs^36–38^.

Hypoxia-inducible factors (HIFs) are transcriptional factors that help cells to adapt to hypoxic conditions. They consist of three main HIFα subunits: HIF1α, HIF2α, and HIF3α, encoded by the genes *HIF1 A*, *HIF2 A* (also called Endothelial PAS Domain Protein 1, *EPAS1*), and *HIF3 A*, respectively, along with a β subunit, HIF1β, encoded by the gene Aryl Hydrocarbon Receptor Nuclear Translocator (*ARNT*)^39–41^. Under normoxia, the HIFα subunits are ubiquitinated for degradation through a process involving the von Hippel-Lindau (VHL) protein and prolyl hydroxylases (PHDs)^42^. When O_2_ is deficient, the HIFα subunits are stabilised, allowing them to bind to HIF1β and translocate into the nucleus to initiate transcription^43^. The three HIFα subunits differ in structure. While HIF1α and HIF2α are well-studied and structurally similar, HIF3α is structurally distinct^44^. HIF1α and HIF2α also exhibit different dynamics under hypoxic conditions. HIF1α responds rapidly to hypoxia, being expressed during acute hypoxia (< 24 h), but its levels decrease as hypoxia persists. In contrast, HIF2α expression increases as HIF1α declines and remains stable in chronic hypoxia (> 24 h)^44,45^. These subunits also contribute to distinct functions. In human endothelial cells, HIF1α primarily regulates metabolic reprogramming, while HIF2α is involved in controlling factors related to angiogenic extracellular signalling and extracellular matrix remodeling^46^. Additionally, in muscle satellite stem cells, HIF2α, but not HIF1α, maintains stemness and inhibits differentiation^47^. Therefore, understanding the dynamics and distinct functions of HIF1α and HIF2α in stem cells could provide deeper insights into how these cells adapt to hypoxia and how they can be better controlled for stemness and regeneration.

In this study, we cultured Muse cells under 1% O_2_ hypoxia and found that HIF2α, rather than HIF1α, played a central role in elevating the Muse cell proportion in BM-MSCs. Hypoxia also elevated pluripotency gene expression via let-7 upregulation and shifted Muse cell metabolism from oxidative phosphorylation (OXPHOS) to glycolysis. These findings demonstrate the potential of hypoxia as an effective method for elevating Muse cell populations while maintaining their pluripotency gene expression. Hypoxia offers a promising approach to generating a larger quantity of Muse cells for future clinical applications.

## Method

### Cell culture

We used human BM-MSCs (LONZA, PT-2501) in this research. The human BM-MSCs were cultured in Minimum Essential Medium Eagle (αMEM, MilliporeSigma, M4526) supplemented with 10% fetal bovine serum (FBS, Hyclone, SH30910.03), 1x GlutaMAX (Gibco, Thermo Fisher Scientific, 35050-061), 1 ng/mL human basic fibroblast growth factor 2 (FGF2) (MiltenyiBiotech, 130-093-840), and kanamycin sulfate solution (Wako, 117–00961). The cells were cultured in a humidified incubator with 5% CO_2_ at 37 °C. The medium was exchanged every 2 days. HeLa cells were maintained in 4.5 g/L glucose Dulbecco’s modified Eagle’s medium (DMEM, Gibco, ThermoFisher Scientific, 11965-092) supplied with 10% FBS, 1 mM sodium pyruvate (Gibco, ThermoFisher Scientific, 11360-070), and kanamycin sulfate.

### Hypoxia culture

The 1% hypoxia condition was achieved using a humidified multi-gas incubator (SANYO) filled with 5% CO_2_ and 94% N_2_ at 37 °C. For subculturing and medium exchange of the hypoxic culture, all experiments were conducted in an O_2_ concentration-adjustable hypoxic chamber with a HEPA filter. The medium was pretreated under 1% O_2_ hypoxic condition for 1 h before use.

### Muse cell sorting by fluorescence activating cell sorting (FACS)

Muse cells were isolated when BM-MSCs reached full confluency. The cells were first incubated with anti-SSEA-3 rat IgM antibody (1:1000, BioLegend, 330302) at 4 °C for 1 h. This was followed by a secondary antibody incubation with fluorescein isothiocyanate (FITC) AffiniPure goat anti-rat IgM (1:100, Jackson ImmunoResearch, 112-095-075) at 4 °C for 1 h. Purified Rat IgM, κ Isotype Control Antibody (BioLegend, 400801) served as a negative control for setting gates. Antibodies were diluted in FACS buffer consisting of 5% bovine serum albumin (BSA), 2 mM EDTA, and FluoroBrite DMEM (Gibco, Thermo Fisher Scientific, A1896701). Finally, Muse cells were collected using a BD FACSAria II SORP Flow Cytometer Cell Sorter (Becton Dickinson) in the purify mode.

### AnnexinV-PI staining

BM-MSCs were stained with SSEA-3 followed by a FITC secondary antibody. As a positive control, HeLa cells were treated with Ultraviolet (UV) for 5 min and then cultured for 2 days. The cells were then stained with Annexin V-APC (Biolegend, 640920) and Propidium Iodide (PI) solution (Invitrogen, P3566) according to the manufacturer’s instructions for 15 min at room temperature in the dark. After staining, the cells were collected and analysed using the CytoFLEX S Flow Cytometer (Beckman Coulter), and the flow cytometry data were analysed with Kaluza Analysis Software (Beckman Coulter).

### SiRNA knockdown

BM-MSCs or FACS-sorted Muse cells were transfected with 25 pmol HIF1 A-siRNA (Ambion), 25 pmol HIF2 A-siRNA (Ambion), or 25 pmol scrambled siRNA (Ambion) by Lipofectamine 3000 (Invitrogen, Thermo Fisher Scientific) in Opti-MEM Reduced Serum Medium (Thermo Fisher Scientific) for 24 h in 35 mm dishes under normoxia following the manufacturer’s instructions.

### Flow cytometry for muse cell ratio analysis

#### Comparison of Muse cell ratio between normoxia and hypoxia

BM-MSCs at passage five were cultured under normoxic or 1% O_2_ hypoxic conditions for two population doubling levels (PDLs, 1 PDL ≈ 3 days). After culturing, cells were collected, stained as described above, and analysed using the CytoFLEX S Flow Cytometer (Beckman Coulter).

#### Examining the effect of HIFs on Muse cell ratio

BM-MSCs treated with scrambled siRNA were cultured under both normoxic and 1% O_2_ hypoxic conditions, while HIF1 A-siRNA and HIF2 A-siRNA-treated BM-MSCs were cultured under 1% O_2_ hypoxia, all for two PDLs. The Muse cell ratio was then assessed through flow cytometry, following the same procedure.

### Reverse transcriptional polymerase chain reaction (PCR) and quantification PCR (qPCR)

To evaluate the knockdown effect of HIF1 A- and HIF2 A-siRNA, siRNA-treated Muse cells were cultured under 1% O_2_ hypoxia for 2 and 4 days. For assessing pluripotency gene expression, Muse cells were cultured in either normal medium or medium supplemented with 200 µM CoCl_2_ for two days. After the incubation, the total RNA was isolated using the RNeasy Mini Kit (QIAGEN, 74106) following the manufacturer’s protocols. The quality and concentration of the total RNA were measured with a NanoDrop One^C^ (Thermo Fisher Scientific). cDNA was generated by reverse transcription PCR using PrimeScript Reverse Transcriptase (Takara Bio., 2680 A) in a Takara Thermal PCR Cycler (Takara Bio.). The primers used in this research are listed in Table 1. For qPCR, we used SYBR Green qPCR Master Mix (Thermo Fisher Scientific, A66732) and the 7500 Fast Real-Time PCR System (Applied Biosciences). The melt curve was confirmed to ensure the specificity of the PCR products. Actin beta (*ACTB)* was used as an endogenous control, and the 2^−ΔΔCT^ relative quantification method was used for all analyses.

**Table 1:** Primers.

Name	Sequence (5’→3’) or assay name	
HK2-F	GAGCCACCACTCACCCTACT	PrimerBank ID: 40806188c1
HK2-R	CCAGGCATTCGGCAATGTG	PrimerBank ID: 40806188c1
PFK1-F	AGCGTTTCGATGATGCTTCAG	PrimerBank ID:266453618c2
PFK1-R	GGAGTCGTCCTTCTCGTTCC	PrimerBank ID:266453618c2
LDHA-F	TTGACCTACGTGGCTTGGAAG	PrimerBank ID: 260099724c2
LDHA-R	GGTAACGGAATCGGGCTGAAT	PrimerBank ID: 260099724c2
PDK1-F	CTGTGATACGGATCAGAAACCG	PrimerBank ID: 37595546c1
PDK1-R	TCCACCAAACAATAAAGAGTGCT	PrimerBank ID: 37595546c1
PDHA1-F	TGGTAGCATCCCGTAATTTTGC	PrimerBank ID: 291084749c1
PDHA1-R	ATTCGGCGTACAGTCTGCATC	PrimerBank ID: 291084749c1
OGDH-F	GGCTTCCCAGACTGTTAAGAC	PrimerBank ID: 259013551c1
OGDH-R	GCAGAATAGCACCGAATCTGTTG	PrimerBank ID: 259013551c1
CS2-F	TGCTTCCTCCACGAATTTGAAA	PrimerBank ID: 38327624c1
CS2-R	CCACCATACATCATGTCCACAG	PrimerBank ID: 38327624c1
IDH2-F	CCCGTATTATCTGGCAGTTCATC	PrimerBank ID: 28178831c2
IDH2-R	ATCAGTCTGGTCACGGTTTGG	PrimerBank ID: 28178831c2
HIF1 A-F	GAACGTCGAAAAGAAAAGTCTCG	PrimerBank ID: 194473734c1
HIF1 A-R	CCTTATCAAGATGCGAACTCACA	PrimerBank ID: 194473734c1
HIF2 A-F	TGACAGCTGACAAGGAGAAGAAA	NCBI Primer-BLAST
HIF2 A-R	AGCTGATTGCCAGTCGCAT	NCBI Primer-BLAST
HIF3 A-F	ATGCGGTCAGCAAGAGCATC	PrimerBank ID: 326807023c2
HIF3 A-R	AGACGATACTCTCCGACTGGG	PrimerBank ID: 326807023c2
ACTB-F	CATGTACGTTGCTATCCAGGC	PrimerBank ID: 4501885a1
ACTB-R	CTCCTTAATGTCACGCACGAT	PrimerBank ID: 4501885a1
POU5 F1-F	GGTGGAGGAAGCTGACAACA	NCBI Primer-BLAST
POU5 F1-R	CTGATCTGCTGCAGTGTGGG	NCBI Primer-BLAST
SOX2-F	TCCAACATCCTGAACCTCAGC	NCBI Primer-BLAST
SOX2-R	TCTGCGTCACACCATTGCT	NCBI Primer-BLAST
NANOG-F	CAGCTCGCAGACCTACATGA	NCBI Primer-BLAST
NANOG-R	CTCGGACTTGACCACCGAAC	NCBI Primer-BLAST
KLF4-F	CACCCACACTTGTGATTACGC	NCBI Primer-BLAST
KLF4-R	TGTTTACGGTAGTGCCTGGTC	NCBI Primer-BLAST

### Western blot

To examine HIF dynamics under 1% O_2_ hypoxia, Muse cells treated with scrambled siRNA, HIF1 A-siRNA, or HIF2 A-siRNA were cultured under 1% O_2_ hypoxia for 0, 2, 4, 8, 24, and 48 hours. For evaluating HIF dynamics in Muse cells exposed to 200 µM CoCl_2_, FACS-sorted Muse cells were directly cultured under 1% O_2_ hypoxia for 0, 4, 8, 24, and 48 hours. After the designated incubation periods, the Muse cells were lysed. To lyse cells cultured on culture dishes, we washed the cells twice with 1x cold phosphate-buffered saline (PBS) and then lysed them with lysis buffer (4% SDS, 20% glycerol, 125 mM Tris-HCl, pH 6.8). The cell lysate was collected using a cell scraper (SPL, 90020) and transferred to a 1.5 mL tube by pipetting. A 26G x 1/2” hypodermic needle (Terumo, NN-2613 S) was used to homogenise the cell lysate, which was then quickly boiled for 5 min. After centrifugation at 13,000 rpm, the supernatant was taken for protein quantification with the Pierce™ BCA Protein Assay Kit (Thermo Fisher Scientific, 23227) following the manufacturer’s instructions. Sodium dodecyl sulfate-polyacrylamide gel electrophoresis (SDS-PAGE) was conducted using SDS-PAGE gels, and proteins were transferred to polyvinylidene difluoride membranes (Millipore, IPVH00010). After antibody reactions, the blots were processed with Pierce ECL Plus Western Blotting Substrate (Thermo Fisher Scientific, 32132). Images were obtained using Fusion FX imaging systems (Vilber). The intensity of the bands was analysed with ImageJ software.

The antibodies used in this study are listed as follows: HIF1α (BD, 610959, 1:1000), HIF2α (Novus Biologicals, NB100-122, 1:2000), and β-actin (Abcam, ab6276, 1:10000).

### Oxygen consumption rate (OCR) and extracellular acidification rate (ECAR)

OCR and ECAR were measured using the Seahorse XFe96 Analyzer (Seahorse Bioscience). We seeded 10,000 cells per well and pre-cultured them for 12 h before measurement. The details and concentrations of the reagents used in this experiment are listed in Table 2 for both OCR and ECAR assays. Measurements of OCR and ECAR in hypoxic cultured cells were conducted in a hypoxic chamber with an O_2_ concentration of 1%.

**Table 2:** Reagents for OCR and ECAR.

Reagents	Stock concentration	Final concentration	Catalogue number
**OCR**			
Calibration solution	—	—	Seahorse, 100840-000
XF Assay medium	—	—	Seahorse, 102353-100
Glucose	2.5M	2.5 mM	Sigma, G7021
L-Glutamine	x100	2 mM	Sigma, G8540
Oligomycin	10 mM/DMSO	1 μM	Sigma, 75351
FCCP	10 mM/DMSO	1 μM	Sigma, C2920
Antimycin	10 mM/DMSO	1 μM	Sigma, A8674
Rotenone	10 mM/DMSO	1 μM	Sigma, R8875
**ECAR**			
Calibration solution	—	—	Seahorse, 100840-000
XF Assay medium	—	—	Seahorse, 102353-100
Glucose	2.5M	2.5 mM	Sigma, G7021
L-Glutamine	200 mM/PBS	2 mM	Sigma, G8540
Oligomycin	10 mM/DMSO	1 μM	Sigma, 75351
2-Deoxy-Glucose	10 mM/DMSO	1 μM	Sigma, D8375

We calculated fundamental parameters such as basal respiration, ATP production, and maximal respiration for OCR, as well as glycolysis, glycolytic capacity, and glycolytic reverse for ECAR, following the manufacturer’s instructions.

### Statistical analysis

All data analyses were conducted using Graphpad Prism 8.0. Data are presented as mean ± SD. The unpaired Student’s t-test calculated the statistical significance of differences between 2 groups. For comparison of more than 2 groups, 1-way ANOVA was conducted (*p < 0.05, **p < 0.01, ***p < 0.001, ns: no significance).

## Results

### 1% O_2_ hypoxia increased the proportion of muse cells in BM-MSCs

**Fig. 1:**
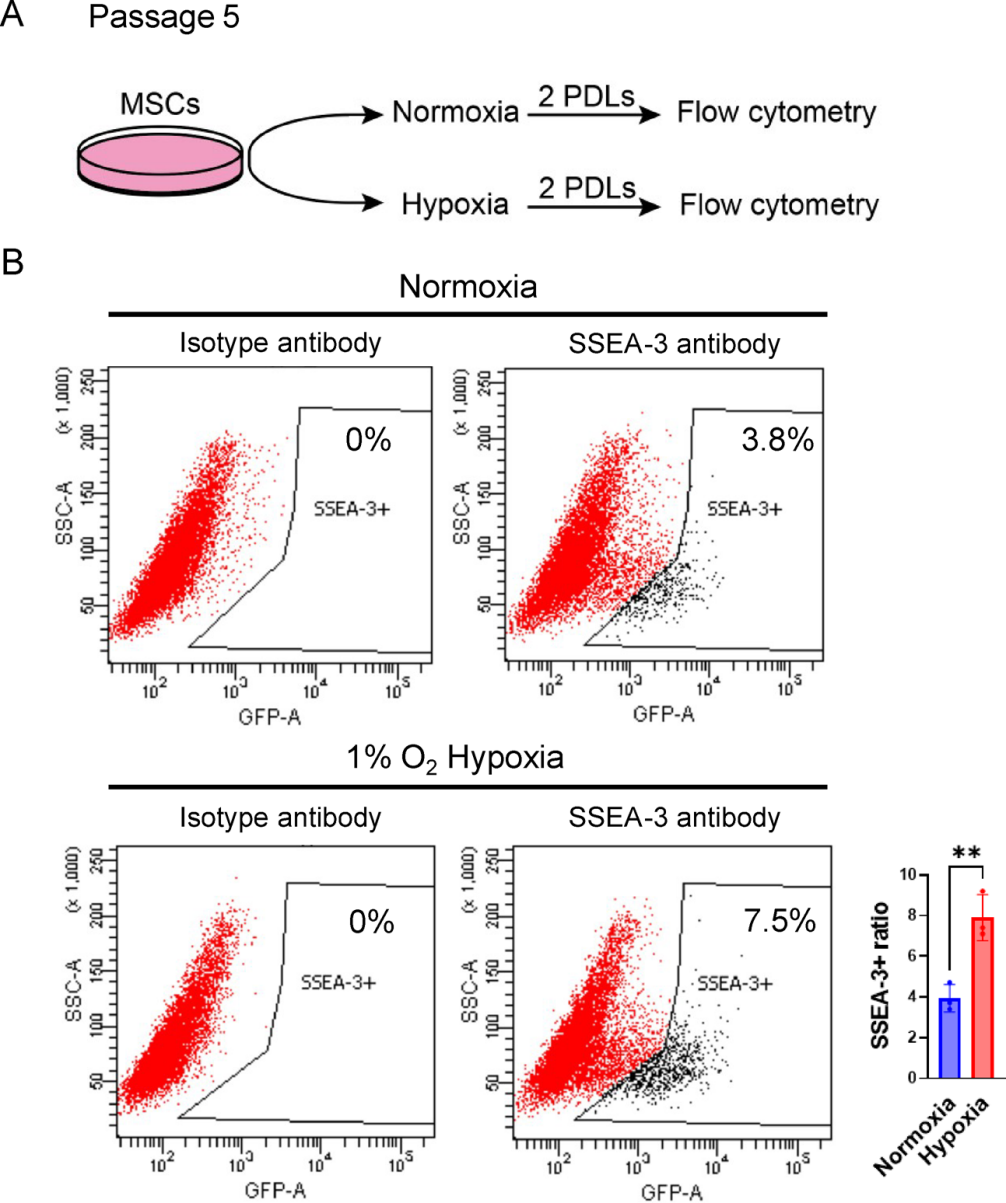
1% O_2_ hypoxia increased the proportion of Muse cells within BM-MSCs. Experimental design for comparing the Muse cell proportion within BM-MSCs. Flow cytometry analysis of Muse cell ratio within BM-MSCs under normoxia and 1% O_2_ hypoxia. An isotype antibody was used for gate-setting control. The average and statistical analysis of three replicates (bar plot). Only one representative set of flow cytometry data is shown. **p < 0.01.

BM-MSCs were cultured in αMEM supplemented with 10% FBS and 1 ng/mL FGF2 under normoxia for 5 PDLs, from passage 1 to 5. At passage 5, BM-MSCs were seeded at 50% confluency and cultured under either normoxia or 1% O_2_ hypoxia for an additional two PDLs. The percentage of SSEA-3(+) Muse cells was then determined using flow cytometry (Fig. 1A). In the flow cytometry, the ratio of Muse cells under normoxia was 3.9 ± 0.68%, while that under 1% O_2_ hypoxia was 7.9 ± 1.34%, suggesting that hypoxia increased the percent of Muse cells nearly twofold with only 2 PDLs (*p* < 0.01) (Fig. 1B). A purified rat IgM antibody was used as a negative control to define the gate for cell sorting^6,9,48–50^ (Fig. 1B). Annexin V/PI staining double staining showed that, under normoxia, most cells (99.28%) were located in AnnexinV and PI double-negative quadrant (lower left quadrant), suggesting viable non-apoptotic cells. The proportion of early apoptotic cells (AnnexinV+/PI-), late apoptotic cells (AnnexinV+/PI+), and necrotic cells (AnnexinV-/PI+) was 0.41%, 0.29%, and 0.02%, respectively (Supplementary Fig. 1 A). After exposure to 1% O_2_ hypoxia, the proportion of viable cells, early apoptotic cells, late apoptotic cells, and necrotic cells were 98.49%, 0.02%, 0.63%, and 0.86%, respectively (Supplementary Fig. 1 A). For positive control, HeLa cells were stained under the same conditions. In comparison to the untreated HeLa cells, where the early apoptotic cells, late apoptotic cells, and necrotic cells were 0.05%, 1.14%, and 3.41%, respectively. UV treatment led to a substantial increase to 1.26%, 72.31%, and 6.67%, respectively (Supplementary Fig. 1B). These results suggested that exposure to 1% O_2_ hypoxia had minimal impact on Muse cell survival while significantly increasing the proportion of Muse cells within BM-MSCs.

### The HIF dynamics in Muse cells under 1% O_2_ hypoxia

**Fig. 2:**
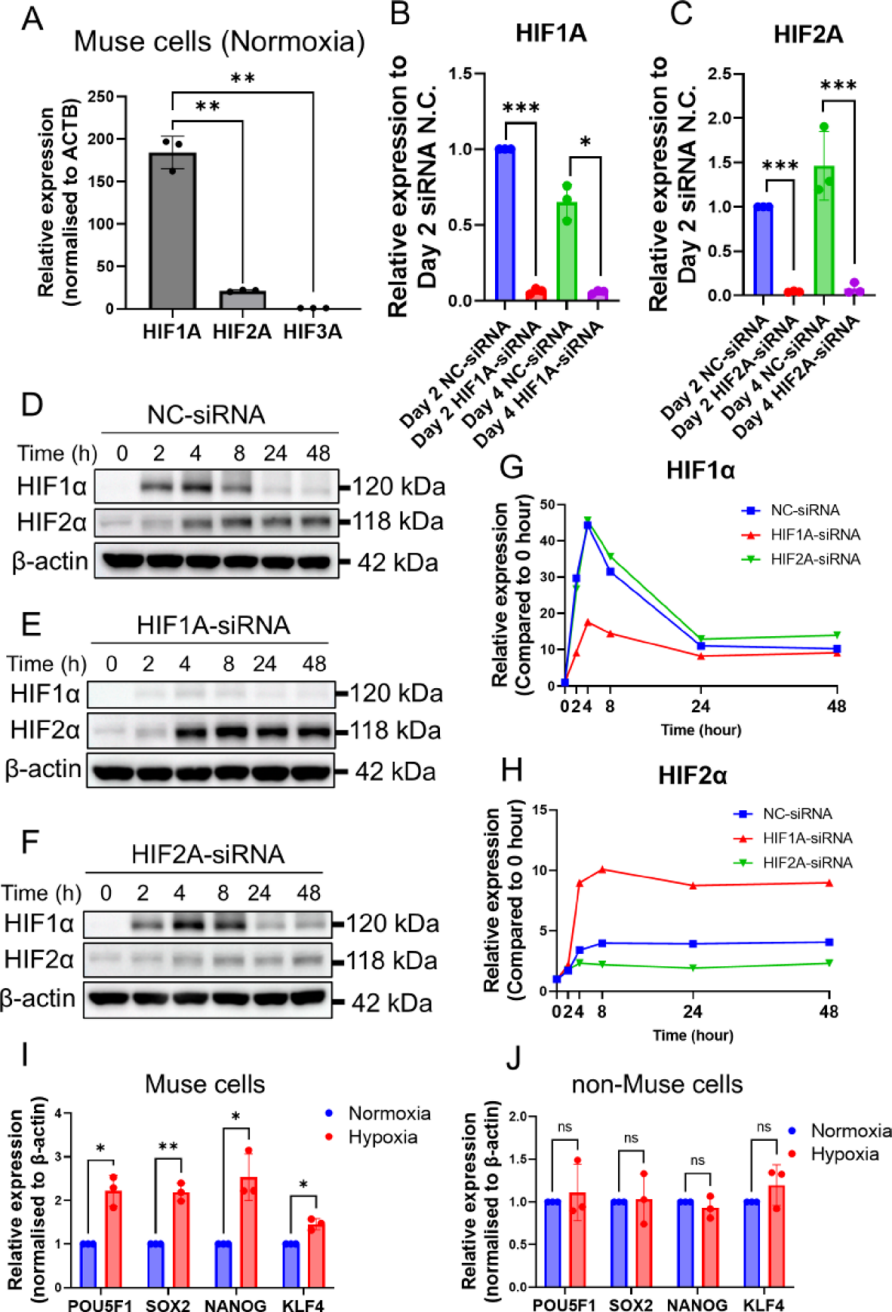
The HIF dynamics in Muse cells under 1% O_2_ hypoxia culture. qPCR comparison of *HIF1 A*, *HIF2 A*, and *HIF3 A* expression in Muse cells under normoxia (*n* = 3 for each). qPCR analysis of *HIF1 A* expression before and after *HIF1 A* knockdown under 1% O_2_ hypoxia (*n* = 3). qPCR analysis of *HIF2 A* expression before and after *HIF2 A* knockdown under 1% O_2_ hypoxia (*n* = 3). Western blot analysis of HIF1α and HIF2α expression in NC-siRNA-transfected Muse cells over time. β-actin was used as an endogenous control. Western blot analysis of HIF1α and HIF2α expression in *HIF1 A*-siRNA-transfected Muse cells over time. β-actin was used as an endogenous control. Western blot analysis of HIF1α and HIF2α expression in *HIF2 A*-siRNA-transfected Muse cells over time. β-actin was used as an endogenous control. Quantification analysis of Western blot comparing HIF1α expression among NC-siRNA-, HIF1 A-siRNA-, and HIF2 A-siRNA-transfected Muse cells. Quantification analysis of Western blot comparing HIF2α expression among NC-siRNA-, HIF1 A-siRNA-, and HIF2 A-siRNA-transfected Muse cells. Comparison of pluripotency gene expression in Muse cells under normoxia and hypoxia (*n* = 3). Comparison of pluripotency gene expression in non-Muse cells under normoxia and hypoxia (*n* = 3). ACTB was used as an endogenous control for normalisation. *p < 0.05, **p < 0.01, ***p < 0.001.

HIF is known to play a critical role in regulating the hypoxic response of cells^51^. We first investigated the expression of HIF subunits in Muse cells. Under normoxia, qPCR analysis revealed that *HIF1 A* and *HIF2 A* expression levels were 185 ± 19.7 (*p* < 0.01) and 21 ± 1.13 times (*p* < 0.01) higher, respectively, than *HIF3 A* in Muse cells (Fig. 2A). Due to the low expression of *HIF3 A*, subsequent experiments focused on *HIF1 A* and *HIF2 A*.

We investigated how HIF regulates the increase in Muse cell proportion within BM-MSCs under hypoxia through loss-of-function experiments, namely, using siRNA to knock down *HIF1 A* and *HIF2 A*. The effect of HIF1 A- and HIF2 A-siRNA was evaluated by using qPCR and Western blot. After introducing siRNAs, Muse cells were cultured under 1% O_2_ hypoxia for 4 days. qPCR showed that the suppression of *HIF1 A* and *HIF2 A* expression was sustained until day 4 (Fig. 2B and C). *HIF1 A* expression showed a significant decrease on day 2 (*p* < 0.001) and day 4 (*p* < 0.05) compared to negative control (NC)-siRNA introduced Muse cells (Fig. 2B). Similarly, *HIF2 A* expression was significantly reduced at both day 2 (*p* < 0.001) and day 4 (*p* < 0.001) compared to NC-siRNA-Muse cells (Fig. 2C).

Given that HIF1α responds to short-term hypoxia (< 24 h), while HIF2α is activated under prolonged hypoxia (> 48 h)^44^, we examined the effects of *HIF1 A-* and *HIF2 A*-siRNAs on their protein expression levels using Western blot analysis across a time course under 1% O_2_ hypoxia. Accordingly, in hypoxia-treated NC-siRNA Muse cells, HIF1α expression increased by 2 h, peaked at 4 h, and then decreased (Fig. 2D and G). In contrast, HIF2α expression increased from 2 h and remained stable for up to 48 h (Fig. 2D and H). After *HIF1 A*-siRNA introduction, HIF2α expression in Muse cells increased more rapidly and reached higher levels compared to that in the NC- siRNA-Muse cells (Fig. 2D and E, and H). In contrast to this, *HIF2 A-*siRNA introduction did not affect the expression of HIF1α in Muse cells (Fig. 2D and F, and G). The original Western blot membranes were shown in Supplementary Fig. 3 A-3D.

These results demonstrated that the effects of HIF1 A-siRNA and HIF2 A-siRNA can persist for up to 4 days and HIF protein revealed a dynamic shift in Muse cells. HIF1α exhibited a rapid increase followed by a decline over the short term, whereas HIF2α showed a slower but sustained increase over the long term. Notably, HIF1α knockdown accelerated the expression of HIF2α in Muse cells.

### Muse cells expressed a higher level of pluripotency genes under 1% O_2_ hypoxia

One of the defining features of Muse cells is their significantly higher expression of pluripotency genes, in contrast to non-Muse cells, which are the SSEA-3-negative subset of MSCs^50,52^. We compared pluripotency gene expression under normoxia and 1% O_2_ hypoxia in both cell types. SSEA-3 (+) Muse and SSEA-3 (-) non-Muse cells were sorted from normoxia-cultured MSCs (Supplementary Fig. 1 C). The non-Muse gate was set at around 40% of MSCs. After sorting, Muse and non-Muse cells were cultured under normoxia and 1% O_2_ hypoxia for two days. In Muse cells, the expression of *POU5 F1* (*p* < 0.05), *SOX2* (*p* < 0.01), *NANOG* (*p* < 0.05), and *KLF4* (*p* < 0.05) was significantly higher under hypoxia than under normoxia (Fig. 2I). However, in non-Muse cells, there was no significant difference between the two conditions (Fig. 2J).

These results suggested that 1% O_2_ hypoxia helped maintain pluripotency gene expression in Muse cells but not in non-Muse cells.

### HIF2α regulated the Muse cell ratio in BM-MSCs

**Fig. 3:**
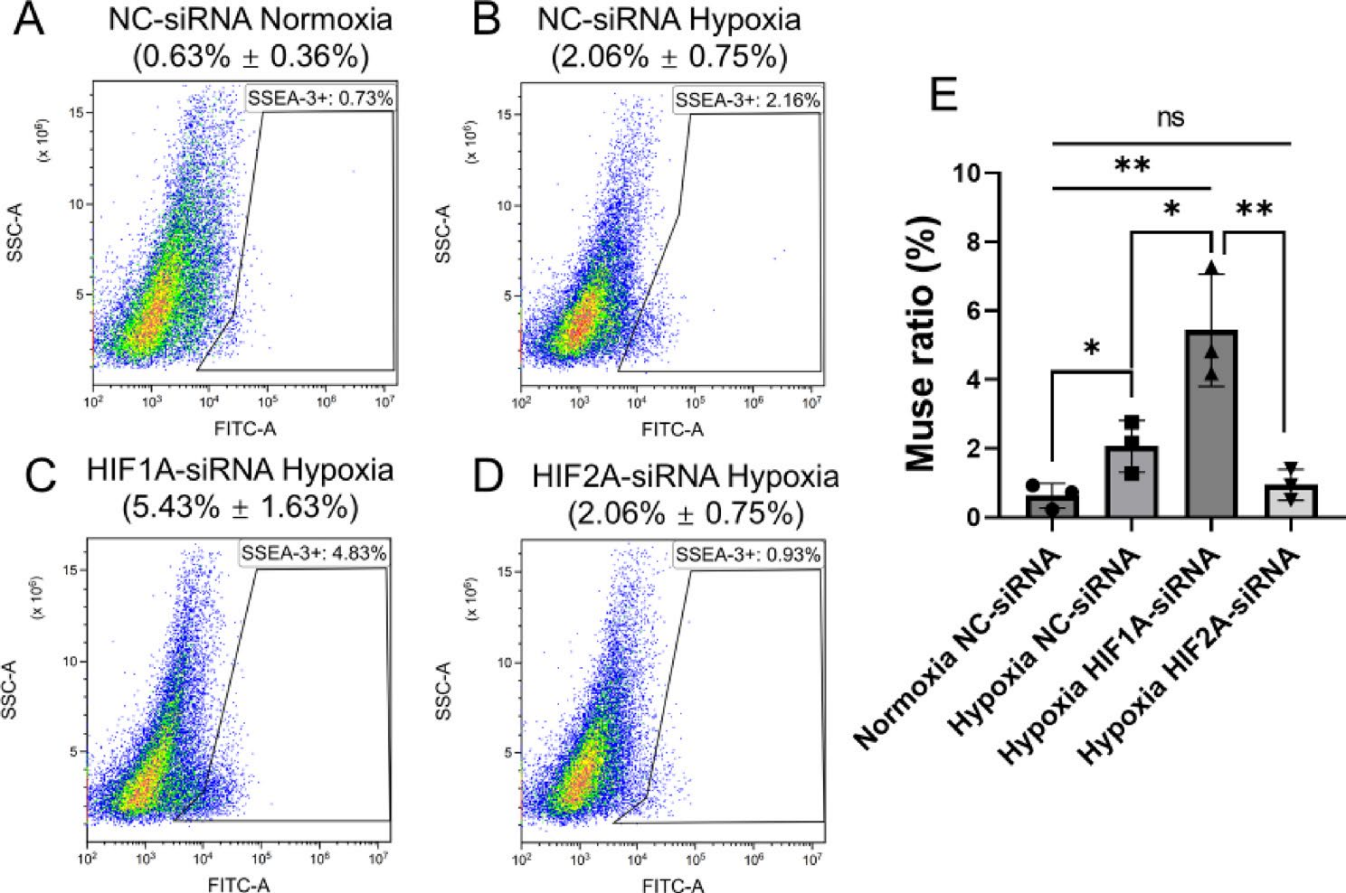
HIF2α regulated Muse cell ratio within BM-MSCs. (**A-D**) Flow cytometry analysis comparing the Muse cell ratio within BM-MSCs. Only one representative set of flow cytometry data is shown on **A-D**. (**E**) Comparison of Muse cell proportion in BM-MSCs among normoxia NC-siRNA, hypoxia NC-siRNA, hypoxia HIF1 A-siRNA, and HIF2 A-siRNA group (*n* = 3). *p < 0.05, **p < 0.01, ns: no significant.

We then investigated the roles of HIF1α and HIF2α in regulating the Muse cell ratio in BM-MSCs by flow cytometry. The results shown in Fig. 3A and D represent data from a single set of measurements, while the labelled mean ± SD values are based on three sets of measurements. Under 1% O_2_ hypoxia, the Muse cell ratio in the NC-siRNA introduced BM-MSCs was 2.06% ± 0.75% (Fig. 3B and E), significantly higher than that observed under normoxia (0.63% ± 0.36%) (Fig. 3A and E, *p* < 0.05). Interestingly, knocking down *HIF1 A* in BM-MSCs cultured under hypoxia further increased the Muse cell population to 5.43%±1.63 (Fig. 3C and E), with a statistically significant increase compared to the hypoxia NC-siRNA introduced BM-MSCs (2.06% ± 0.75%, Fig. 3B and E, *p* < 0.05). However, knocking down HIF2α prevented the increase in the Muse cell ratio under hypoxic conditions, resulting in 0.93% ± 0.45 of BM-MSCs with no significant difference from normoxia NC-siRNA introduced BM-MSCs (Fig. 3A, D and E).

These results suggested that HIF2α, rather than HIF1α, played a critical role in increasing the proportion of Muse cells in BM-MSCs under 1% O_2_ hypoxia.

### Hypoxia shifted the Muse cell metabolism from OXPHOS to Glycolysis

Muse cells were sorted from BM-MSCs and cultured under either normoxic or 1% O_2_ hypoxic conditions. Their metabolic activity was assessed by measuring the OCR, an indicator of OXPHOS, and ECAR, which reflects glycolytic activity. Hypoxia increased ECAR and decreased OCR compared to the normoxic condition in Muse cells (Supplementary Fig. 2 A and 2B). We compared the expression of glycolysis- and OXPHOS-related genes between normoxia and hypoxia. Glycolysis-related genes, lactate dehydrogenase A (*LDHA*) and pyruvate dehydrogenase kinase 1 (*PDK1*), measured by qPCR, significantly increased by 1- and 2-fold under hypoxia. OXPHOS-related genes (*PDHA1*) and citrate synthase 2 (*CS2*) significantly decreased compared to normoxia, while no significant changes were observed in hexokinase 2 (*HK2*), oxoglutarate dehydrogenase (*OGDH*), and isocitrate dehydrogenase 2 (*IDH2*) in Muse cells. However, the expression of glycolytic gene phosphofructokinase 1 (*PFK1*) decreased (Supplementary Fig. 2 C).

These findings suggest that hypoxia shifts the metabolism of Muse cells from OXPHOS to glycolysis-dominant, with the key glycolysis-regulating genes *LDHA* and *PDK1* upregulated.

### HIF1α maintained glycolysis-dominant metabolism

**Fig. 4:**
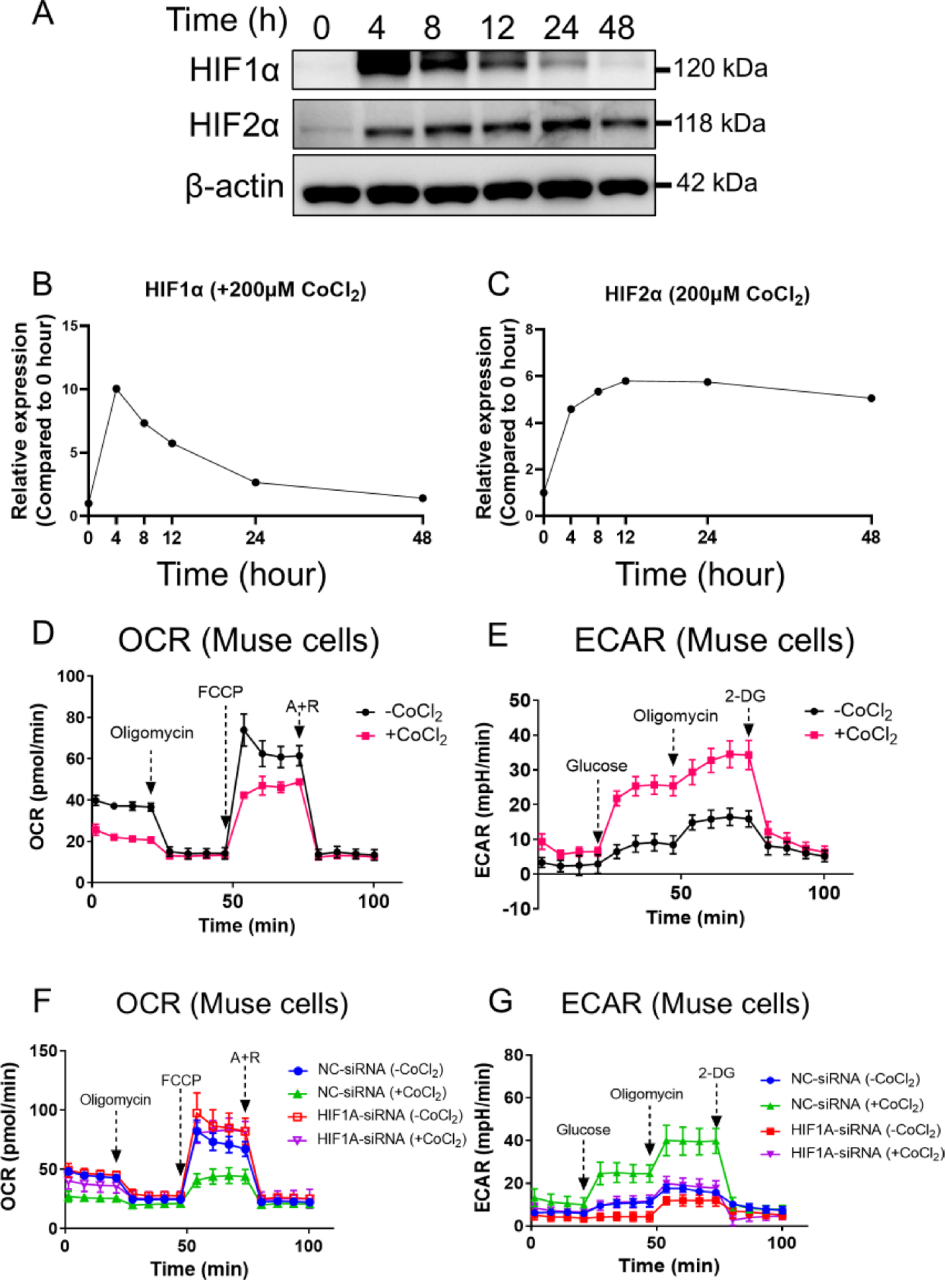
Hypoxia mimicked by CoCl_2_ treatment in Muse cells. (**A-C**) Western blot analysis of HIF1α and HIF2α expression in Muse cells over time (up). Quantification analysis of Western blot (down). β-actin was used as an endogenous control. Comparison of OCR before and after 200 µM CoCl_2_ treatment (*n* = 5). Comparison of ECAR before and after 200 µM CoCl_2_ treatment (*n* = 5). Comparison of OCR before and after *HIF1 A* knockdown in Muse cells (*n* = 15). Comparison of ECAR before and after *HIF1 A* knockdown in Muse cells (*n* = 5).

Next, to assess the function of HIF1α in the metabolism of Muse cells, CoCl_2_, known to inhibit the degradation of HIF, was used to mimic HIF-mediated hypoxia^53^. After adding 200 µM CoCl_2_, the expression of HIF1α and HIF2α showed similar trends to those observed under 1% O_2_ hypoxia: the HIF1α expression peaked at 4 h and then returned to the baseline level by 48 h (Fig. 4A and B), while the HIF2α expression increased at 4 h and maintained the level until 48 h (Fig. 4A and C). The original Western blot membranes were presented in Supplementary Fig. 4A−C. Additionally, Muse cells treated with CoCl₂ for 8 h showed a decreased OCR and an increased ECAR compared to the untreated control group (Fig. 4D and E), similar to the response observed under 1% O_2_ hypoxia (Supplementary Fig. 2 A and 2B). Due to the similar metabolic trends observed between 1% O_2_ hypoxia and CoCl_2_-mimicked hypoxia, we opted to use CoCl_2_-mimicked hypoxia for the next experiment.

Using Muse cells, the CoCl_2_-treated NC-siRNA group (green) showed a decreased OCR and increased ECAR compared to the CoCl_2_-untreated NC-siRNA group (blue) (Fig. 4F and G). However, after *HIF1 A* knockdown, CoCl_2_ treatment (purple) did not largely change the OCR and ECAR levels compared to the CoCl_2_-untreated *HIF1 A*-siRNA group (red) (Fig. 4F and G).

These results indicate that HIF1α is pivotal in maintaining glycolysis-dominant metabolism.

### CoCl_2_ upregulated pluripotency gene expression through upregulating let-7

**Fig. 5:**
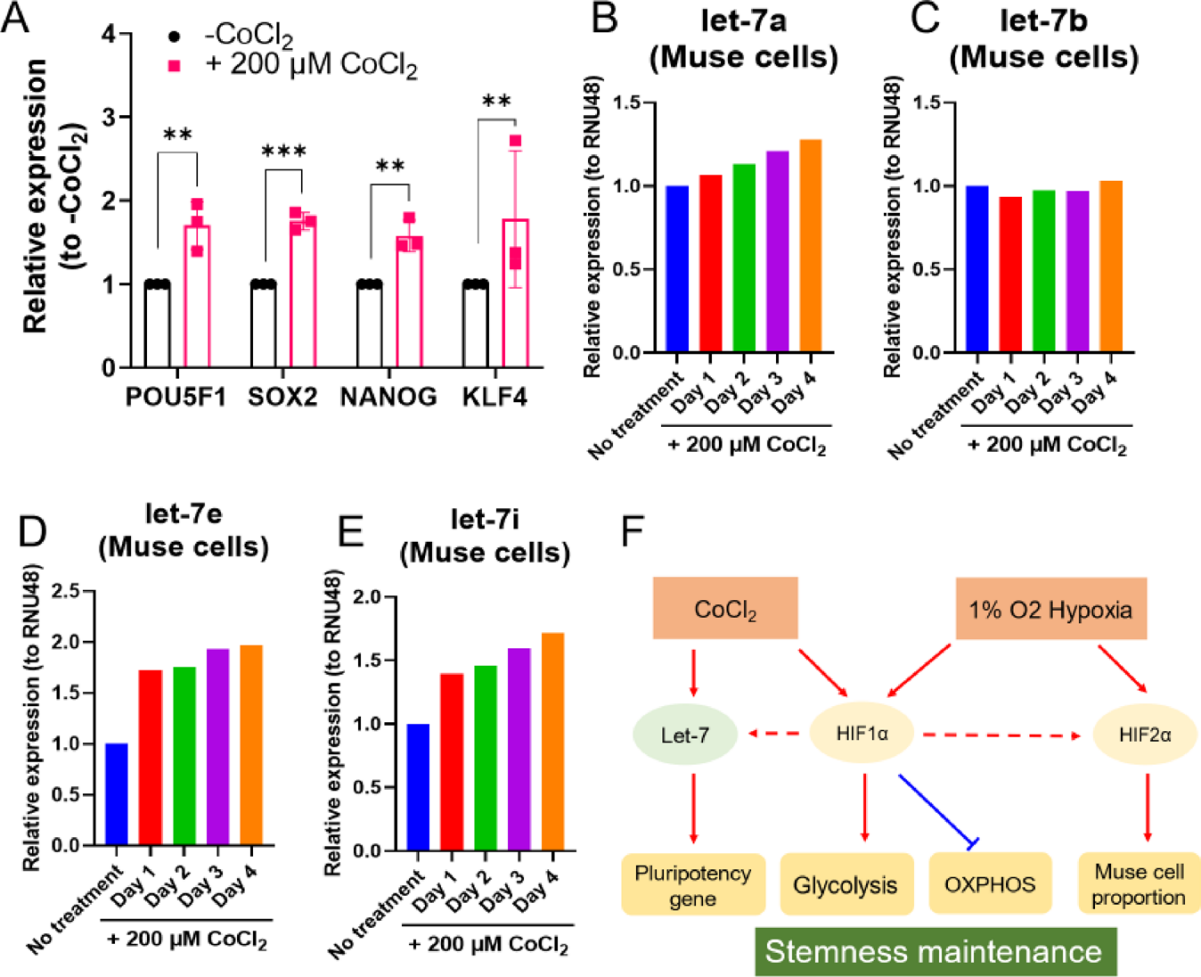
CoCl_2_ increased pluripotency gene expression through upregulating let-7 expression. The effect of CoCl_2_ treatment on pluripotency gene expression (*n* = 3). The effect of CoCl_2_ treatment on let-7a expression. The effect of CoCl_2_ treatment on let-7b expression. The effect of CoCl_2_ treatment on let-7e expression. The effect of CoCl_2_ treatment on let-7i expression. Schematic summary of this research. **p < 0.01, ***p < 0.001.

We investigated the effect of 200 µM CoCl_2_ treatment on the expression of pluripotency genes. qPCR analysis revealed that, in Muse cells, the treatment with 200 µM CoCl_2_ for 2 days upregulated the expression of *POU5 F1*, *SOX2*, *NANOG*, and *KLF4*, compared to the untreated (Fig. 5A). Let-7 has been reported to maintain the expression of pluripotency genes, such as *POU5 F1*, *SOX2*, *NANOG*, and *KLF4*, in Muse cells^9^. We used qPCR to analyse the expression of let-7a, let-7b, let-7e, and let-7i, identified as the highly expressed members of the let-7 family in Muse cells^9^. We then treated Muse cells with 200 µM CoCl_2_ and analysed the expression of let-7 by qPCR. Compared to the CoCl_2_-untreated control group, the expression of let-7a, let-7e, and let-7i gradually increased from day 1 to day 4 after 200 µM CoCl_2_ treatment (Fig. 5B and D, and E), except let-7b, which remained unchanged (Fig. 5C).

These results showed that CoCl_2_, known to mimic hypoxic conditions, can upregulate pluripotency gene expression, possibly by increasing the expression of let-7a, let-7e, or let-7i.

## Discussion

This study suggested that 1% O_2_ hypoxia increased the proportion of Muse cells in BM-MSCs by around two-fold, along with the upregulation of HIF2α (Figs. 1B and 3). CoCl_2_ treatment upregulated pluripotency gene expression, likely mediated by the increased expression of let-7, which was known to maintain pluripotency gene expression in Muse cells (Fig. 5)^9^. Additionally, HIF1α regulated the metabolic shift from OXPHOS to glycolysis, a key feature of stem cell metabolism, where glycolysis predominates to support the maintenance of stemness across various stem cell types (Fig. 4F and G and Supplementary Fig. 2). These changes suggested that hypoxic conditions effectively upregulated their proportion in BM-MSCs while maintaining pluripotency. A summary of the proposed schema was shown in Fig. 5F.

Knockdown of HIF1α elevated HIF2α expression (Fig. 2E), further increasing the Muse cell proportion in BM-MSCs under 1% O_2_ hypoxia (Fig. 3C and E). The detailed molecular mechanism underlying the relation between the upregulation of HIF2α expression and Muse cell proportion in BM-MSCs needs to be investigated in the future. The MEK/ERK pathway is shown to maintain the proportion of Muse cells in BM-MSCs^9^. Hypoxia stimulated the MEK/ERK pathway, involving HIF2α in this process^54,55^. Thus, the hypoxia-induced increase in Muse cells might have been mediated through HIF2α activation of the MEK/ERK pathway. Compared to HIF1α and HIF2α, HIF3α has been less extensively studied. In Muse cells, although the expression level of HIF3α is much lower than that of HIF1α and HIF2α (Fig. 2A), it may still contribute to the hypoxic response. Our studies have demonstrated that HIF1α and HIF2α play significant roles in regulating metabolic processes and the proportion of Muse cells within MSCs. We believe these two factors sufficiently explain the key responses observed within our experiments. Investigating how HIF3α regulates Muse cell behaviour under prolonged hypoxia would be an interesting point for future research.

It was noteworthy that the Muse cell ratio in BM-MSCs differed between Fig. 1B (3.8%) and Fig. 3A (0.73%). While Muse cells usually comprise several percent of MSCs under normoxia, the culture conditions of MSCs significantly influenced the Muse cell proportion. The quality of FBS, culture medium composition, the concentration of FGF2 in the culture medium, and the quality of MSC donors can influence the Muse cell ratio^9^. In the experiment shown in Fig. 3A, BM-MSCs were first transfected with siRNA, followed by culturing in Opti-MEM Reduced Serum Medium for 24 h, and then reverted to the complete culture medium for BM-MSCs (αMEM, 10%FBS, and 1 ng/mL FGF2) before isolating Muse cells. In the experiment shown in Fig. 1B, on the other hand, BM-MSCs were continuously maintained in the complete culture medium and then isolated Muse cells. The culture condition for siRNA transfection conditions required low serum without FGF2 in Opti-MEM. Since FGF2 was shown to be one of the critical factors for the survival and proliferation of Muse cells in BM-MSCs^9^, the culture condition for siRNA transfection might have made it difficult for Muse cells to maintain their proportion in BM-MSCs. Despite these variations, our results consistently demonstrated that 1% O_2_ hypoxia effectively increased the Muse cell ratio in BM-MSCs.

Expanding isolated Muse cell numbers while simultaneously maintaining their stemness/pluripotency was considered an ideal approach for obtaining an abundant supply of Muse cells in a time-efficient and cost-effective manner. In this study, alongside the increase in Muse cell proportion induced by 1% O_2_ hypoxia, CoCl_2_-mimicked hypoxia also elevated pluripotency gene expression (Fig. 5A). This effect may be attributed to the upregulation of let-7a, let-7e, and let-7i expression following CoCl_2_ treatment (Fig. 5B and D, and E), as let-7 was shown to maintain the expression of pluripotency genes in Muse cells^9^. In fact, HIF1α was known to bind to the hypoxia-response element (HRE) in the promoter region of let-7, leading to the upregulation of let-7 expression^56^. In this manner, HIF upregulation might have evaluated the expression of pluripotency genes through the upregulation of let-7 in Muse cells.

An interesting observation is that 1% O_2_ hypoxia increased pluripotency gene expression in Muse cells but not in non-Muse cells (Fig. 2I and J). This may be attributed to the fact that pluripotency gene expression is inherently much lower in non-Muse cells compared to Muse cells^50^. Previous studies have shown that HIF2α can bind to the *Oct4* promoter and enhance its expression in mouse embryonic stem cells^57^. It has also been suggested that HIF2α binds to the HRE in the *NANOG* promoter, contributing to regulating pluripotency under hypoxic conditions in human embryonic stem cells^58^. These findings indicated that in Muse cells, HIF might similarly bind to the promoters of pluripotency genes to help maintain their expression under hypoxia. In contrast, this regulatory mechanism was likely ineffective in non-Muse cells due to their low baseline expression of pluripotency genes. Further studies are needed to elucidate the underlying mechanisms.

Acute severe hypoxia at 1% O_2_ levels was known to induce apoptosis in human ESCs, whereas moderate hypoxia at 5% O_2_ levels did not^59,60^. In this study, 1% O_2_ hypoxia did not induce apparent apoptosis in Muse cells (Supplementary Fig. 1). Due to their stress-resistant properties and their original location in the BM, where O_2_ concentration could be as low as 1.3%, Muse cells were assumed to be able to adapt to low oxygen^24,61^.

1% O_2_ hypoxia and CoCl_2_ treatment shifted the metabolism of Muse cells from OXPHOS to glycolysis, mainly mediated by HIF1α (Supplementary Fig. 2 and Fig. 4D and G). ROS generated from OXPHOS led to oxidative stress, inducing senescence and decreased proliferation activity of MSCs^62,63^. The senescence of MSCs also led to the depletion of the Muse cell pool in MSCs^9^. Therefore, high OXPHOS may not be an optimal strategy for Muse cells to maintain their stemness/pluripotency.

Muse cells are known to be constantly mobilised from the BM, the main reservoir of Muse cells in vivo, to the peripheral blood and are supplied to each organ^4^. Upon tissue damage, they are known to be mobilised into the peripheral blood, as reported in patients with acute myocardial infarction and stroke, probably in order to repair damaged tissues since they are known to be endogenous reparative stem cells^5,17,64^. Once in the peripheral blood, Muse cells are exposed to a higher O_2_ concentration than that in the BM. After homing to the damaged tissue, they encounter a toxic and stressful microenvironment with varying oxygen levels, where they differentiate into tissue-constituent cells to replace damaged/apoptotic cells by a phagocytosis-dependent mechanism and facilitate tissue repair^64,65^. A metabolic shift is known to precede differentiation^66^. Therefore, transitioning the microenvironment from the BM to peripheral blood may promptly shift Muse cell metabolism from a pluripotent/glycolytic state to a differentiation/OXPHOS state, making them ready to repair the tissue through differentiation. In fact, Muse cells under normoxic conditions exhibit OXPHOS dominant, lower HIF-1α and let-7 levels, and decreased pluripotency gene expression compared to those under hypoxic conditions (Fig. 5F). This state resembled that of differentiated cells more than the pluripotent-like state.

In this study, we discovered that hypoxia effectively increased the proportion of Muse cells in BM-MSCs while simultaneously enhancing pluripotency gene expression. Hypoxia shifted cellular metabolism from an OXPHOS/differentiation state to a glycolysis/stemness state, demonstrating that hypoxia may help the maintenance of stemness/pluripotency of Muse cells. While underlying mechanisms need to be investigated in the future, the hypoxic condition is suggested to be one of the feasible approaches to efficiently collect Muse cells without gene introduction.

## Electronic supplementary material

Below is the link to the electronic supplementary material.

Supplementary Material 1

## Data Availability

All data and supporting information are contained in the article. Data is available upon reasonable request by contacting the corresponding author.
