# Supplementary material for: Hypoxia boosts pluripotent-like muse cell ratio in mesenchymal stromal cells and upregulates the pluripotency gene expression
Source: Sci Rep. 2025 Aug 25;15:31183. doi: 10.1038/s41598-025-03806-x (PMC12378945; doi:10.1038/s41598-025-03806-x)
Supplement: Supplementary file 1 — Supplementary Material 1 [file 41598_2025_3806_MOESM1_ESM.docx]

**
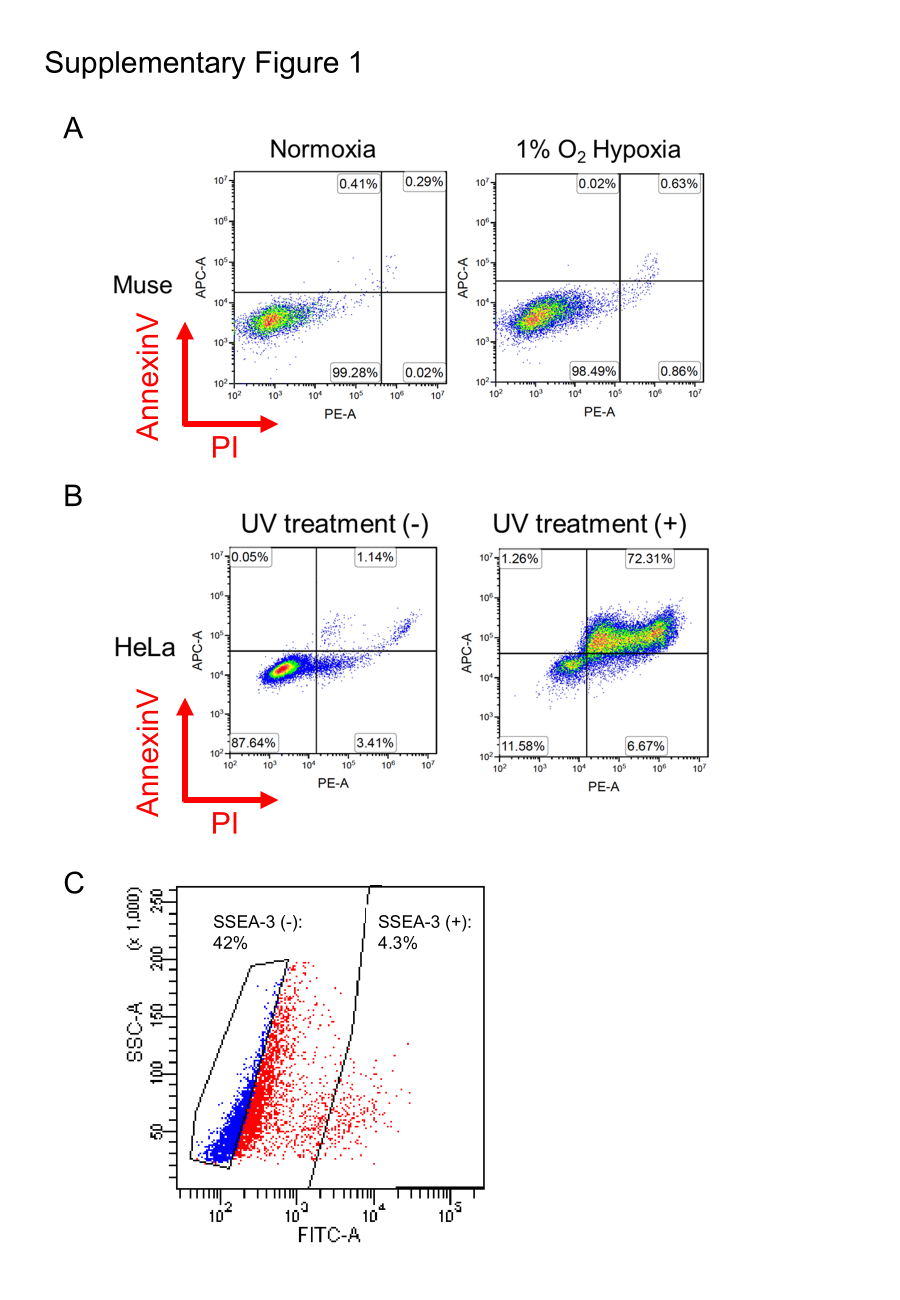
**

**Supplementary Figure 1. 1% O_2_ hypoxia did not induce apoptosis in Muse cells.**

1. Flow cytometry analysis of apoptosis under normoxia or 1% O_2_ hypoxia using AnnexinV-PI staining.
2. Flow cytometry analysis with Annexin V-PI staining was conducted on UV-treated HeLa cells, which were used as a positive control.
3. Gating strategy for sorting Muse and non-Muse cells.

**
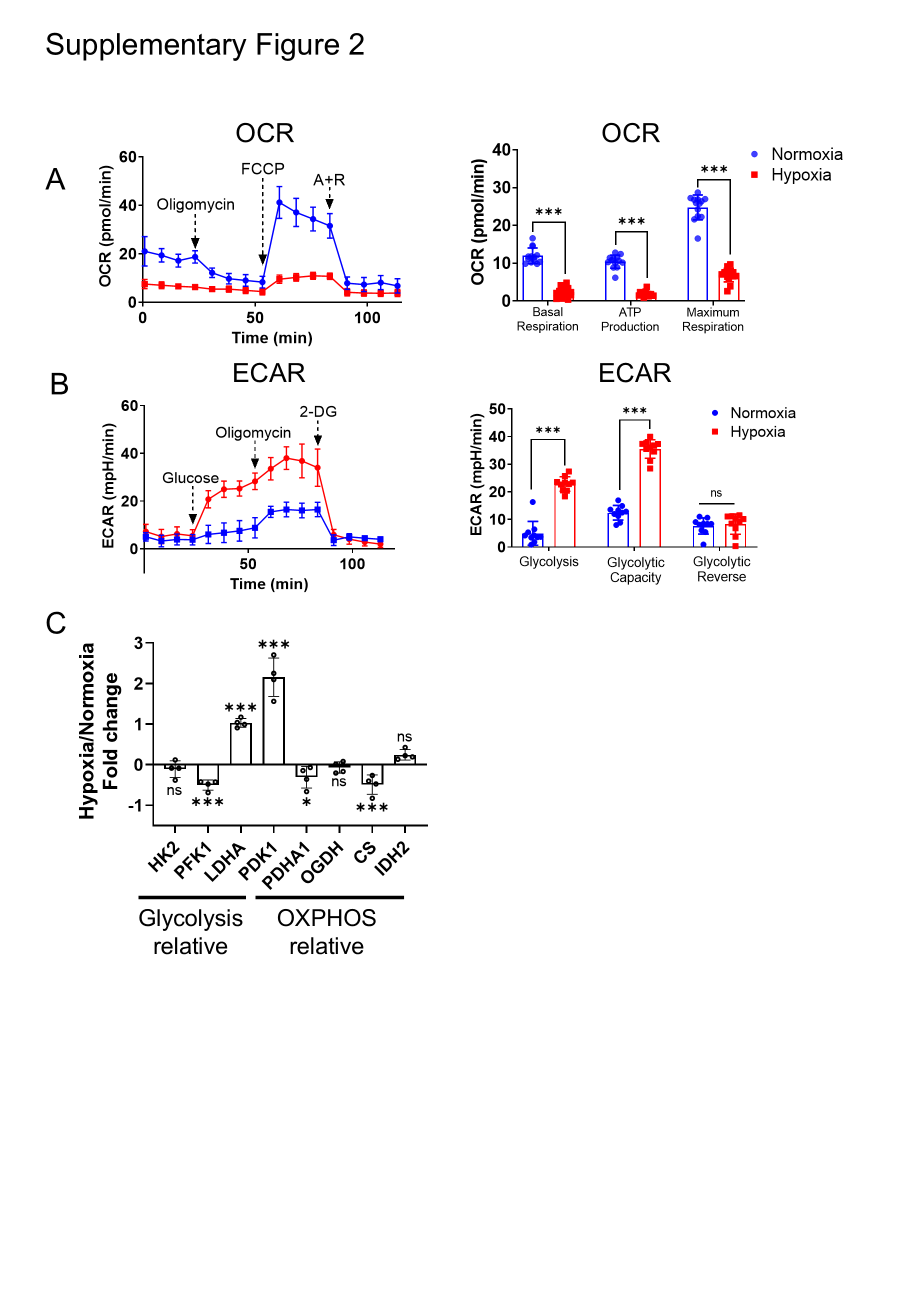
**

**Supplementary Figure 2. Hypoxia caused the metabolism of Muse cells to shift from OXPHOS to glycolysis.**

1. Comparison of OCR in Muse cells under normoxia and 1% O_2_ hypoxia culture (n=10 for both conditions).
2. Comparison of ECAR in Muse cells under normoxia and 1% O_2_ hypoxia culture (n=10 for both conditions).
3. qPCR comparison of glycolysis- and OXPHOS-related gene expression in Muse cells under normoxia and 1% O_2_ hypoxia (n=3 for both conditions).

* p<0.05, ***p<0.001, ns: no significant.

**
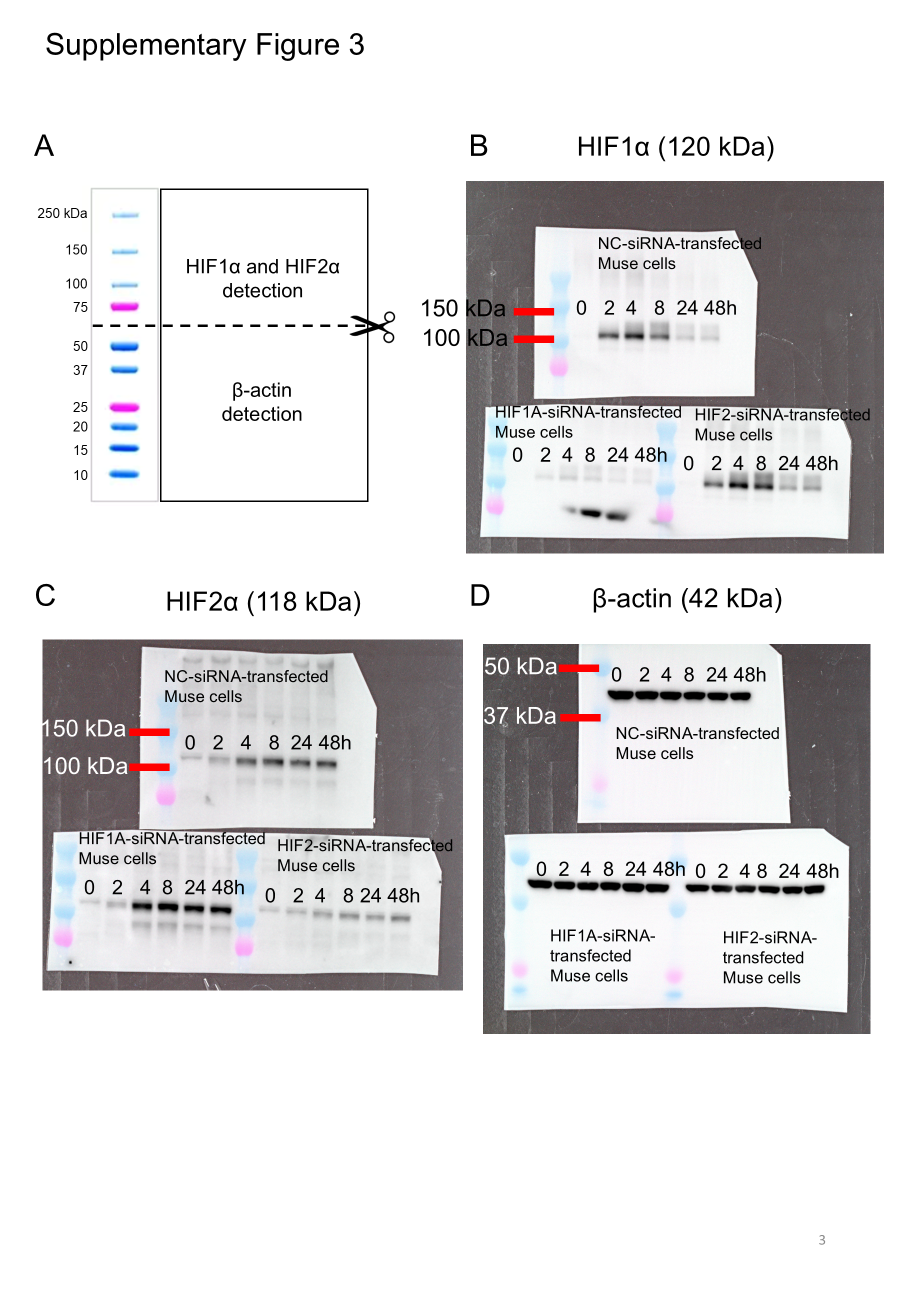
**

**Supplementary Figure 3. Original Western blot membranes supporting the data in Figure 2.**

1. Membranes were cut between 50 kDa and 75 kDa to detect HIFs and β-actin.
2. Original Western blot membranes for detecting HIF1α.
3. Original Western blot membranes for detecting HIF2α.
4. Original Western blot membranes for detecting β-actin.


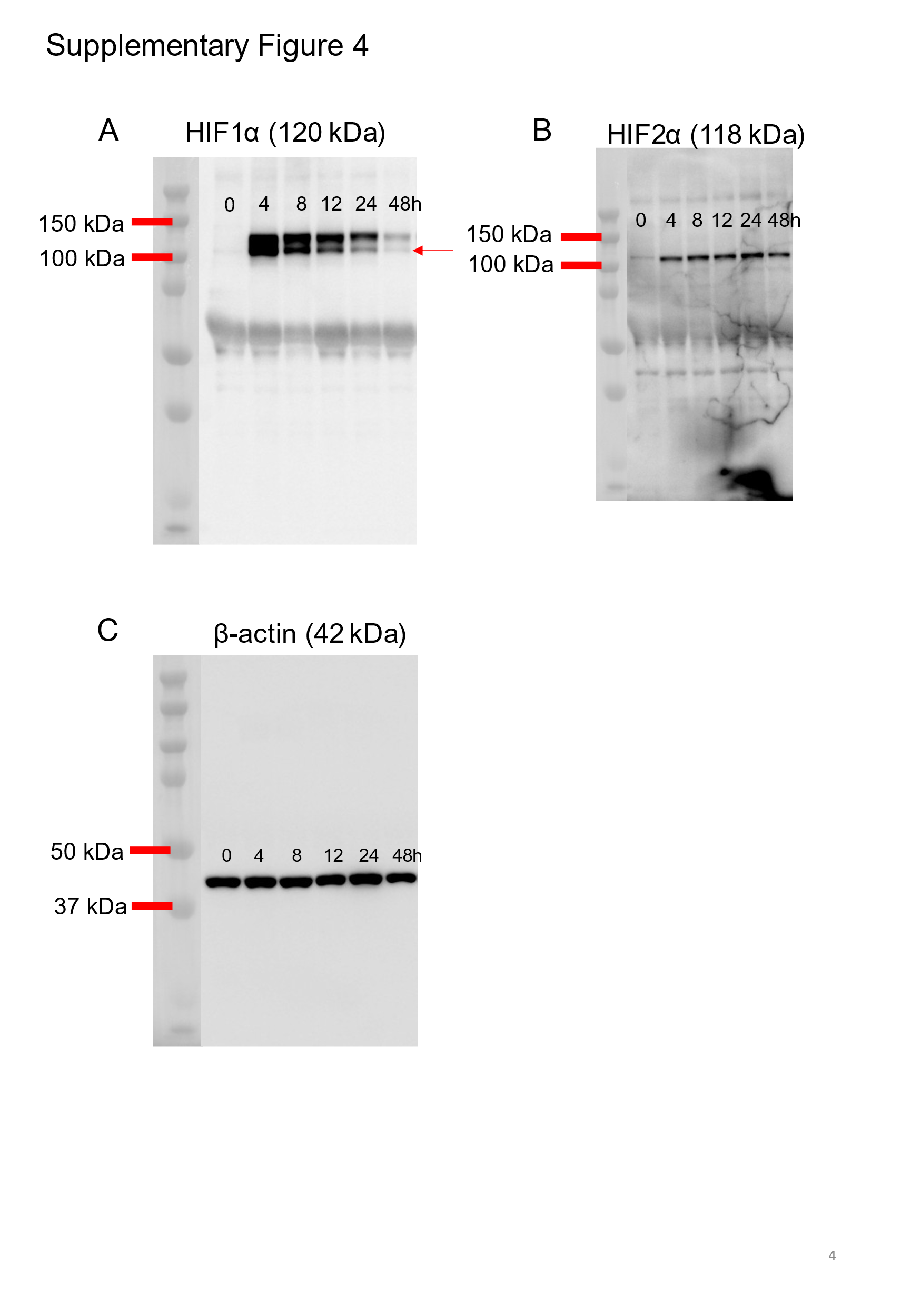


**Supplementary Figure 4. Original Western blot membranes supporting the data in Figure 4A.**

1. Original Western blot membranes for detecting HIF1α in Figure 4A.
2. Original Western blot membranes for detecting HIF2α in Figure 4A.
3. Original Western blot membranes for detecting β-actin in Figure 4A.
